# Segregation GWAS to linearize a non-additive locus with incomplete penetrance: an example of horn status in sheep

**DOI:** 10.1186/s12711-024-00928-0

**Published:** 2024-09-03

**Authors:** Naomi Duijvesteijn, Julius H. J. van der Werf, Brian P. Kinghorn

**Affiliations:** 1 Cooperative Research Centre for Sheep Industry Innovation, Armidale, NSW 2351 Australia; 2 School of Environmental and Rural Science, University of New England, Armidale, NSW 2351 Australia; 3 Present Address: Hendrix Genetics Research, Technology & Services B.V., P.O. Box 114, 5830 AC Boxmeer, The Netherlands

## Abstract

**Background:**

The objective of this study was to introduce a genome-wide association study (GWAS) in conjunction with segregation analysis on monogenic categorical traits. Genotype probabilities calculated from phenotypes, mode of inheritance and pedigree information, are expressed as the expected allele count (EAC) (range 0 to 2), and are inherited additively, by definition, unlike the original phenotypes, which are non-additive and could be of incomplete penetrance. The EAC are regressed on the single nucleotide polymorphism (SNP) genotypes, similar to an additive GWAS. In this study, horn phenotypes in Merino sheep are used to illustrate the advantages of using the segregation GWAS, a trait believed to be monogenic, affected by dominance, sex-dependent expression and likely affected by incomplete penetrance. We also used simulation to investigate whether incomplete penetrance can cause prediction errors in Merino sheep for horn status.

**Results:**

Estimated penetrance values differed between the sexes, where males showed almost complete penetrance, especially for horned and polled phenotypes, while females had low penetrance values for the horned status. This suggests that females homozygous for the ‘horned allele’ have a horned phenotype in only 22% of the cases while 78% will be knobbed or have scurs. The GWAS using EAC on 4001 animals and 510,174 SNP genotypes from the Illumina Ovine high-density (600k) chip gave a stronger association compared to using actual phenotypes. The correlation between the EAC and the allele count of the SNP with the highest –log10(p-value) was 0.73 in males and 0.67 in females. Simulations using penetrance values found by the segregation analyses resulted in higher correlations between the EAC and the causative mutation (0.95 for males and 0.89 for females, respectively), suggesting that the most predictive SNP is not in full LD with the causative mutation.

**Conclusions:**

Our results show clear differences in penetrance values between males and female Merino sheep for horn status. Segregation analysis for a trait with mutually exclusive phenotypes, non-additive inheritance, and/or incomplete penetrance can lead to considerably more power in a GWAS because the linearized genotype probabilities are additive and can accommodate incomplete penetrance. This method can be extended to any monogenic controlled categorical trait of which the phenotypes are mutually exclusive.

**Supplementary Information:**

The online version contains supplementary material available at 10.1186/s12711-024-00928-0.

## Background

Genome-wide association studies (GWAS) for apparently monogenic traits can be obscured by complex genetic models including effects of dominance, epistasis, sex dependent gene expression, and incomplete penetrance. An example of such a trait is the polled-horn phenotype in sheep. A variety of horn phenotypes occurs within various sheep breeds and this can be sex-dependent. Horn phenotypes in sheep are generally represented in three groups, horned, scurred or knobbed (abnormal horn development) and polled (absence of any horn formation). The genetic background of the horn phenotype has been discussed for many years and is still under debate. The first hypothetical model proposed for the inheritance in sheep was nominated by Dolling [1], with three alleles controlling horn formation, which are Ho+ (normal horns), HoL (sex-limited horns), and HoP (polled), which they had compared with a model with two loci, each with two alleles. Their proposed model was also confirmed by Coltman and Pemberton [2]. Montgomery et al. [3] were the first to perform a linkage analysis using microsatellites and they showed horn status is controlled by a single locus on OAR 10 (OAR for *Ovis aries*). Following studies further narrowed down to a region near the gene *RXFP2* [4–6]. Finally the region was fine-mapped to a 1.78-kb insertion in the 3ʹ-UTR of the *RXFP2* gene [7].

The quantitative trait locus (QTL) on OAR10 explains a large proportion (~ 76%) of the heritable genetic variation for horn size and base circumference in Soay sheep [5]. Genome-wide association studies so far have not found any other significant signals besides tagging the insertion at OAR10, indicating that the horn phenotypes could be controlled by a single locus. However, the *RXFP2* insertion does not fully explain the phenotype, as reported by Lühken et al. [8] and Duijvesteijn et al. [9]. Different models have been investigated, especially in breeds which have variable horn status and/or where differences in expression between sexes are observed, e.g., in Merino and in breeds from Southern Europe and Africa, and phenotype patterns seem not fully consistent with a single locus model [8]. Currently, the most accepted mode of inheritance of horns is additive in ewes and complete dominance in rams [5]. In further studies on Merinos, statistical evidence for sex-dependent differences in the additive and dominance effect for horned and polled phenotypes was provided [9], although the model still did not fully explain the variation in phenotypes observed. A possible explanation could be incomplete penetrance, where not all sheep carrying the ‘horn-causing’ genotype show the associated phenotype (horns). In the example of horns, assuming only one locus involved, the level of penetrance would indicate the maximum prediction accuracy that could be achieved using genomic information for prediction of the phenotype.

A method based on segregation analysis described by Kerr and Kinghorn [10] was extended to detect a single locus mutation that determines a categorical trait with incomplete penetrance [11]. Information from pedigree and phenotypes is first used to calculate genotype probabilities at a putative QTL, given starting assumptions on allele frequency and pattern of penetrance (the probability of each horn phenotype within each QTL genotype). These genotype probabilities are then used to estimate allele frequency, and together with the phenotypes, to estimate the pattern of penetrance. This process is iterated until convergence is reached.

These genotype probabilities, when expressed as the probable number of alleles carried, or expected allele count (EAC, range 0 to 2), are inherited additively, by definition, unlike the original phenotypes, which are non-additive, categorical, and potentially of incomplete penetrance. This means that a classical additive GWAS on the probabilities could increase power to detect single nucleotide polymorphisms (SNPs) that are associated with the phenotype compared to a GWAS on the original phenotypes.

The objective of this study is to introduce a method that performs a GWAS in conjunction with segregation analysis on monogenic categorical traits. We apply this method to real horn phenotypes using a genetic model which includes parameters for sex-dependent expression, dominance, and incomplete penetrance, and these parameters can be estimated. A GWAS is then run on the EAC. We also used simulation with a known causative mutation to test the extent in which incomplete penetrance causes prediction error in Merino sheep for horn status.

## Methods

### Phenotypes

Background information on phenotype collection and genotypes has been given in more detail in Duijvesteijn et al. [9]. Briefly, 4001 Merino sheep born between 2007 and 2011 from eight research flocks have horn status recorded (Table 1). All male sheep where castrated at marking (six weeks of age) and before horn scoring which was postweaning, at around four months of age. The pedigree consisted of 12,482 animals with 186 sires and 2733 dams having progeny with phenotypes.

**Table 1:** Number of observed phenotypes for male and female Merinos

Horn status	Female	Male (wether)
Polled	1123	511
Knobs/scurs	1237	561
Horned	88	481
Total	2448	1553

### Use of genotype probabilities

In this section we describe a method to find the best-fitting genotype–phenotype model, calculating QTL genotype probabilities assuming a monogenic inheritance with incomplete penetrance for a categorical trait (step 1). A genome-wide association analysis regressing the probable number of QTL alleles on individual SNP genotypes was used to identify markers that can predict horn status (step 2). Step 2 is expected to show increased power compared to traditional genome-wide association analysis where observed phenotypes are regressed on individual SNP genotypes, because, unlike the phenotypes, the genotype probabilities are linear and could accommodate information about incomplete penetrance.

#### Step 1: calculate genotype probabilities

In first instance, genotype probabilities for genotypes aa, Aa or aA, and AA at a single putative QTL were calculated for all animals, females only or males only, based on horn phenotypes (Table 1), pedigree, and starting values for penetrance using the program Geneprob, which implements the method of Kerr and Kinghorn [10]. We will refer to these as genotype 0 for aa, genotype 1 for Aa and aA and genotype 2 for AA.

In a subsequent Maximization-step, penetrance values (example in Table 2) from each of the three QTL genotypes to each horned phenotype were estimated conditional on the genotype probabilities, as well as allele frequencies. We iterated with these expectations and maximisation steps to converge on penetrance values, allele frequency and genotype probabilities.

**Table 2:** Starting penetrance values for a gene with incomplete penetrance

Phenotype^a^	Genotype class		
	0	1	2
0 = Polled	1.00	0.25	0.00
1 = Scurs/knobs	0.00	0.75	0.00
2 = Horned	0.00	0.00	1.00

^a^Polled = absence of horn formation, scurs/knobs = abnormal formation of horn, horned = normal horn development

After convergence, each animal has three genotype probabilities, and a genotype probability index (GPI) as calculated based on Kinghorn [12]. The GPI, ranging from 0 to 100 %, is a measure of utility of these probabilities for an individual. Animals whose genotypes are known with complete certainty have a GPI of 100 and animals with no useful information to infer genotype have probabilities equal to Hardy–Weinberg frequencies and a GPI of 0. The mean GPI across the population is a simple measure of utility. The relationship between mean GPI and the correlation between true and predicted genotype status is suggested to be approximately linear [12].

Following convergence of Geneprob, the estimated genotype probabilities were used to calculate the EAC using the following equation:

$$EAC=0*p\left(aa\right)+1*p\left(Aa\right)+1*p\left(aA\right)+2*p\left(AA\right),$$

where $p(x)$ is the genotype probability for the genotype class $x$. The value of EAC lies between 0 and 2, similar to a SNP genotype.

#### Step 2: Genome-wide association study (GWAS)

For the GWAS, EAC was regressed on the SNP genotypes. Similar to a traditional GWAS, − log10(p-values) can be plotted to indicate a possible QTL region. All GWA analyses were performed for four subsets which were (i) only females, (ii) only males, (iii) a combined dataset with EAC values from a segregation analysis on a combined dataset or (iv) a combined dataset with EAC values from separate analyses of the data sets on males and females. Correlations between the allele count of the most significant SNP and the EAC were then calculated to indicate a minimal value for the underlying correlation between putative QTL and phenotype.

For comparison, we also included the results of the GWAS where the original phenotypes were used. The original phenotypes were used for two GWAS. First, horned (0) and non-horned (1) animals were classified and secondly, polled (0) and non-polled (1) animals were classified. These binary phenotypes where regressed on the SNP genotypes using a logistic function. These classifications were chosen in order to not have to assume that knobs or scurs are an intermediate phenotype between horned and polled. Results from these analyses have been published in an earlier study [9].

### Genotypes

In total 293 sheep were genotyped with the Illumina-Ovine 12k, 3708 sheep were genotyped with the OvineSNP50 BeadChip and 454 sheep were genotyped with the Illumina Ovine HD (HD). Quality control per chip were the same; individual SNP genotype records were removed if the average SNP call rate was less than 90%, the GC (GenCal) score was less than 0.6, strong deviation from Hardy–Weinberg equilibrium (χ^2^ > 600), the minor allele frequency < 0.01 and only autosomes were selected. The 12K chip resulted in 11,377 SNPs, the 50K chip in 48,559 SNPs and the HD in 510,174 SNPs after applying the quality control. Genotypes from the 12K chip animals were imputed up to 50K using Beagle software v3.2 [13] and the 50K genotypes were imputed up to HD using FImpute v2.2 [14], and for both programs default settings were used. For both imputation steps, all data from genotyped animals from INF and the Sheep Genomics Flock were used [15, 16], to increase imputation accuracy (22,684 animals for 50k and 2450 for HD). Accuracy of imputation was tested elsewhere [15, 16] and was generally high (average imputation accuracy was 0.98).

### Simulation

Simulation was used to estimate the maximum correlation that can be achieved between a causative mutation and the EAC given varying levels of penetrance. PopSNP, as described by van Eenennaam and Kinghorn [17] was used for simulating SNP data into the existing pedigree (see paragraph Phenotypes). One chromosome was simulated with a founding population size of 100 and a burn-in of 100 generations. One thousand candidate bi-allelic SNPs (alleles 1 and 2) were generated of which 50% of loci have only a single mutation in the whole population (all animals genotype 0, except for a single mutant individual with genotype 1). Those loci are candidates as QTL. After the burn-in, a gene-drop through the real pedigree is performed, where in our case one randomly chosen segregating SNP is selected as the causative mutation. The minor allele frequency of the causative mutation was on average 0.49. Ten replicates were performed where no linkage was simulated between SNPs to guarantee independence across replicates. A phenotype is assigned randomly to each individual conditional on the penetrance matrix estimated from the real phenotypes of the animals in the pedigree. Three different sets of penetrance matrix values (Table 3) were used to investigate its effect on the sensitivity of the correlation between the EAC and the simulated true causative mutation. For example, using scenario 2, an animal with genotype 0 has 100% assignment of phenotype 0, where animals with genotype 1 will have a 25% chance to be assigned phenotype 0 and 50% chance to be assigned phenotype 1 and a 25% chance to be assigned phenotype 2 and a 100% assignment of phenotype 2 with genotype 2 (Table 3). Genotype probabilities for three phenotype classes (polled, knobs/scurs and horned) were calculated per individual after Geneprob converged. The correlation between the allele count at the true causative mutation and the EAC derived from the genotype probabilities is a measure of the maximum correlation that can be expected for a certain degree of penetrance. Average correlation across replicates will be reported with standard error. A lower correlation for the allele count of a predictive SNP would indicate that the SNP is not in full LD with the causative mutation. The correlation should approximately match the average (across replicates) Genotype Probability Index (GPI) as the relationship is approximately linear [12].

**Table 3:** Simulated penetrance values for each genotype class for each of the three phenotype classes for three scenarios

Phenotype value	Scenario1			Scenario2			Scenario3		
	Genotype values			Genotype values			Genotype values		
	0	1	2	0	1	2	0	1	2
Phenotype 0	1.00	0.00	0.00	1.00	0.25	0.00	0.50	0.25	0.00
Phenotype 1	0.00	1.00	0.00	0.00	0.50	0.00	0.50	0.50	0.50
Phenotype 2	0.00	0.00	1.00	0.00	0.25	1.00	0.00	0.25	0.50

## Results

Estimated penetrance matrices are reported in Table 4 for males, females and the combined dataset for horned phenotypes. Note that these results involve no use of SNP genotype data. Results were stable using different sets of starting values. The results show clear differences between males and females. Males with genotype 2 will be horned, while for females with genotype 2 only 22% will be horned and otherwise have scurs or knobs. All males and females with genotype 0, will be polled. Knobs and scurs are more observed in females, where genotype 1 will result in knobs and scurs in 92% of the females. The combined analysis shows an intermediate pattern, where genotype 1 and 2 will give 50% knobs and scurs and 50% horns, where genotype 0 will give a polled phenotype.

**Table 4:** Predicted penetrance values for each genotype class for each of the three phenotype classes and datasets

Phenotype value	Males			Females			Males and females		
	Genotype values			Genotype values			Genotype values		
	0	1	2	0	1	2	0	1	2
Polled	1.00	0.23	0.00	1.00	0.07	0.00	1.00	0.48	0.00
Knobs/Scurs	0.00	0.76	0.00	0.00	0.92	0.78	0.00	0.52	0.59
Horned	0.00	0.01	1.00	0.00	0.02	0.22	0.00	0.00	0.41

**Fig. 1:**
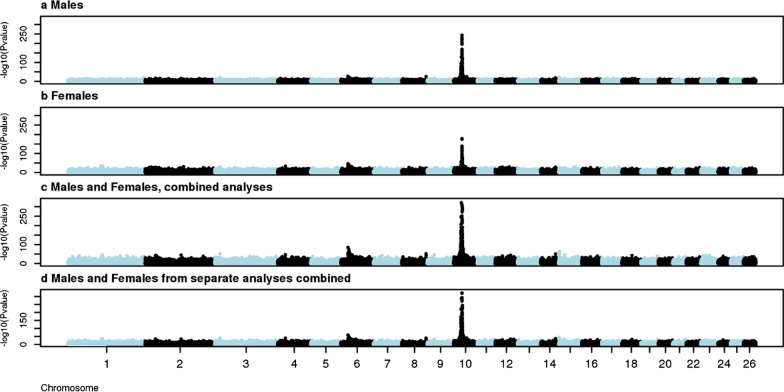
Genome-wide association for the EAC. **a** males (N = 1553), **b** females (N = 2448), **c** combined analyses of males and females together (N = 4001) and **d** combined outputs from the separate males and females analyses (N = 4001). The different chromosomes are indicated with different iterating colors. The significance in –log10(p-value) is indicated on the y-axis

The results of GWAS for EAC are shown in Fig. 1. The most significant SNP for males, females and the combined dataset was OAR10_29546872.1 (rs426516358) when the EAC were regressed on the SNP genotypes. The significance of SNP OAR10_29546872.1 resulted in a level beyond the smallest number possible to report (− log10P values > 321) for all analyses, except for the GWAS for females only of which the − log10 p-value was 178. To be able to further discuss the model fit using EAC, the R^2^ of the model was used to determine which SNP explained the most variance of the model. The highest R^2^ value was 0.53 in males, 0.44 in females, 0.53 in the combined analyses and 0.47 in the analyses combining the separate male and female analyses.

To compare the method using a binary trait (horned/non-horned or polled/non-polled) with the newly proposed method using segregation analysis, we can compare the results from the different GWAS. The GWAS for the binary traits are presented in Additional file 1 Figure S1 and Additional file 2 Figure S2. The most significant SNP was either OAR10_29546872.1 or OAR10_29458450 (rs426516358). The two SNPs are 0.5 Mb apart, located upstream of the insertion and in high LD (r^2^ = 0.985). The significance levels (– log10 p-value) achieved were 206, 17 and 169 (males, females and combined dataset respectively) for horned/non-horned and 122, 224 and 260 (males, females and combined dataset respectively) for polled/non-polled. The signal where genotypes were associated with the EAC (newly proposed method) was notably larger than in any of the GWAS using the phenotypes. Especially in females, where incomplete penetrance values are high, the significance of the association using the EAC was much higher (− log10 P-value of 178 compared to 17 when using actual phenotypes).

Correlations between the most significant SNP (real data), or the true simulated causative mutation using the penetrance matrix obtained from real data (Table 4), and the EAC are reported in Table 5.

**Table 5:** Correlation between the EAC and the most significant SNP, and the mean genotype probability index (GPI), for the real and simulated data split by males and females and males and females combined

		Males	Females	Combined
Real data	Correlation EAC	0.73	0.67	0.73
	Mean GPI	87.77	43.93	41.57
Simulated data^a^	Correlation EAC	0.95 (0.002)^b^	0.89 (0.002)	0.82 (0.002)
	Mean GPI	92.7 (0.21)	80.09 (0.78)	67.1 (0.39)

*EAC* expected allele count

^a^Penetrance values used are shown in Table 4, obtained from the real data

^b^Standard error in brackets

Correlations for the real data are consistently lower than from simulated data. The dataset where only phenotypes of the females were used, showed the lowest correlation using the real data. The correlation for the combined dataset is higher than might be expected from the low mean GPI involved. However, this might reflect the wider range in phenotypes, and hence genotype probabilities, across the two sexes. Moreover, when the QTL was simulated, the smallest correlation was observed in the combined dataset. The maximum correlation in simulated data was 0.95 in males using penetrance values as reported in Table 5. The correlations obtained from the real phenotypes with the causative mutation are consistently lower than the correlation using the EAC. Especially in females, where being horned has a small chance even when the genotype probability for genotype 2 is high (Table 4), the correlation between the real phenotype and true causative mutation was close to zero (0.006). Using the EAC, which incorporates non-additivity and incomplete penetrance, the correlation for females is much higher (0.89).

Correlations for the three additional penetrance values for the three phenotype classes are reported in Table 6. Obviously when complete penetrance values (scenario 1) were simulated, correlation between the EAC and true causative mutation was 1. The more the penetrance values deviated from 1, the lower the correlation between the true causative mutation and the EAC.

**Table 6:** Correlation between the EAC and true causative mutation and mean genotype probability index (GPI) for the simulated data using varying penetrance values

Scenario^a^	Correlation EAC–SNP	Mean GPI
1	1 (0.0)	100 (0.0)
2	0.89 (0.002)	80.00 (0.30)
3	0.66 (0.004)	47.30 (0.57)

^a^The penetrance values for each scenario are shown in Table 3

*EAC*  expected allele count

## Discussion

Incomplete penetrance values per sex were estimated and indicated clear differences between males and females for horn status in sheep. Furthermore, a model which assumed a monogenic inheritance with incomplete penetrance for a categorical trait (horned phenotype), was highly associated with the most significant SNP. However, the correlation between the EAC and the most significant SNP was lower than the correlation resulting from a scenario where the true causative mutation was modelled. This is expected where the most significant SNP is not the true causative mutation and/or where the mutation it marks is not the only genetic influence on the phenotypes observed.

### Methodology

The methodology used in this study to detect the QTL underlying a categorical monogenic trait with incomplete penetrance consists of two steps: first, the EAC based on pedigree and phenotypes is established, followed by running a GWAS. We showed increased significance in detecting the associated SNP (e.g. − log10 p-values of the segregation analyses were larger than when using the real phenotypes). Due to the categorical nature of the trait, performing a GWAS is less efficient, as linearity between classes is quickly assumed or data is split in binary traits. Both are not optimal; therefore, we present a new and alternative method where all data can be used without making assumptions on linearity between the classes of the phenotype.

This study shows the results for the horn phenotype in sheep, but the method could be applied to other monogenic categorical traits affected by incomplete penetrance, which are widely present in other livestock species [18, 19] and humans [20]. Using the proposed ‘Segregation GWAS’ method to determine the most likely causative mutation and to help determine the prediction accuracy that can be expected given incomplete penetrance could be valuable information when providing genetic testing.

Optimization of this method where the segregation analyses and the GWAS inform each other could result in more power to detect the QTL. A further development, given that the trait is affected by sex, could be to categorize the trait by sex and horn status, in which horn status of males and females inform each other. Even though the method used in this study already shows good potential to perform a GWAS for a trait with a mutually exclusive phenotype affected by one locus, improvements could be made which were beyond this study. One path to explore further is the monogenic assumption in the inheritance model. One possible explanation of incomplete penetrance is the effect of modifier genes. Segregation analysis based on a monogenic model but a simulation model with more causative loci might test the sensitivity of our approach to the monogenic assumption of segregation analysis. It is interesting to note here that the GWAS based on EAC revealed a second peak on chromosome 6. Although much smaller than the peak on chromosome 10, with a probability that is orders of magnitude bigger, it is significant and was not seen in a regular GWAS study [9]. Therefore, it is possible that our method is also powerful in detecting segregation at any modifier loci.

The question has arisen whether the current procedure is prone to creating secondary peaks due to some form of artifact. The EAC score is calculated without any dependence on genotypic data. This means that, as for normal traits such as body weight, any artifacts are due to phenomena such as linkage disequilibrium in the genetic markers within and/or across chromosomes. Accordingly, such secondary peaks for EAC are argued to be as important as they might be for GWAS on standard trait data.

One possibility for further investigation is to correct the EAC scores for the main peak effect, then re-run the GWAS to see the impact on the secondary peak. This correction could be done by estimating the effect on EAC at the main peak for each animal, according to its genotypes, and subtracting that from its EAC score.

Further study with additional causative loci acting under various plausible genetic models could demonstrate any power to pick up modifier loci, but also investigate the possibility of the segregation method leading to more false positives due to linkages across the genome.

### Penetrance values related to sex and *RXFP2*

The level of penetrance reflects the proportion of individuals in a given population with a specific ‘horn-causing’ genotype that expresses the corresponding horn phenotype. Reduced or incomplete penetrance indicates the proportion within the population of animals carrying the horn genotype and not expressing the corresponding horn phenotype. Discussed mechanisms underlying incomplete penetrance are modifier genes, copy number variants, age, sex and others. Especially in the case of the horned phenotype in Merino sheep where large differences between sexes are observed, incomplete penetrance due to the influence of sex is interesting. Low penetrance values in women for hypokalemic periodic paralysis compared to men have been observed due to different effects of sex hormones [21]. The discussed underlying mechanism is differential gene regulation in males and females, specifically in relation to sex steroid-responsive genes [22, 23]. Horn formation in sheep is influenced by hormones and environment (seasonal change stimulates horn development). Castrated sheep (wethers) will stop the formation of horns once castrated [24]. A study in Soay sheep observed a similar pattern where 1-day-old lambs were castrated and horn development was similar to those of females [25]. This suggests that sex hormones play a role in horn development. The role of the gene *RXFP2*, where the causative mutation (1.8-kb insertion) is mapped to [7], is to function as a receptor for hormones associated with male primary sex characteristics. Mutations in the *RXFP2* gene have been associated with human cryptorchidism, indicating its involvement in sex-related phenotypes. Therefore, we hypothesize that different expression levels of *RXFP2* between ewes, rams and wethers, could cause different sizes and possibly also formations of horns (scurs and knobs). Although, we recognize that the presence of the insertion (homozygous, genotype class 0) in Merino sheep, is to the best of our knowledge, resulting in a polled phenotype, reflected by the penetrance values reported in Table 4 (full penetrance). Genotype classes different than 0, result in variable horn status per sex due to reasons discussed.

### Real vs. simulated QTL

The results of this study indicated that the model where incomplete penetrance is included, resulted in a high association between the EAC and the best-fitting SNP. However, the correlation was not one. This best-fitting SNP is known to tag the 1.7-kb insertion, but it is not in the insertion itself. Therefore, linkage disequilibrium (LD) between the insertion and the SNP OAR10_29546872.1, could be less than one. Previously published estimates of LD depended on the animals sequenced, and the LD estimated as R^2^ was 0.49 based on 72 sequenced Merino animals [9]. Similar low values for breeds with variable horn status and possible sex-dependent horn phenotype were observed in Lühken et al. [8], where R^2^ between a SNP on the intron of the *RXFP2* gene (OAR10_29511510.1) and the insertion was 0.194. In breeds from completely polled or horned breeds, the value was much higher with R^2^ = 0.635. In a breed which is polled (e.g. Poll Dorset, N = 10) the R^2^ was 1.

These relatively low R^2^ values could explain why the correlation between the real data with the SNP genotype was so much lower than the correlation from the simulated true causative mutation (0.73 vs. 0.95 for males and 0.67 vs. 0.89 for females respectively). The ratio between the correlations (0.73/0.96 and 0.67/0.89) was very similar for males and females (0.76 and 0.75 for males and females respectively), which could indicate a common ‘absence’ of a better predictor (e.g., the insertion). A more complete picture of the role of penetrance in the genetic model would have been obtained if the samples used in this study had been assayed for genetic variance that target the actual genetic variants in the *RXFP2* gene, but this was not possible due to the time since sample collection. It would be interesting in future work to employ our proposed method in data that includes these genotypes along with phenotypic data.

Influence of other genes has been tested before, and was not found to be likely [9], where association studies for polled/non-polled and horned/non-horned correcting for the SNP OAR10_29546872.1, did not show any other region to be significantly associated. Similar to cattle, where a range of mutations in a region on chromosome 1 cause polledness, sheep polledness could also be caused by different mutations in the region of *RXFP2*. This is also discussed by Lühken et al. [8], where in some breeds the 1.8-kb insertion was not causative for the polledness. As noted earlier, our current method showed some evidence of at least one additional region that could be involved, i.e., on chromosome 6, and the GWAS based on genotype probabilities potentially reveals more clearly such regions with modifier genes. Whether that could explain some of the differences between breeds in penetrance could be further explored with data that includes more detailed genotype information in the actual *RXFP2* region.

### Implications

Given the different penetrance values for males and females, and without a method to properly handle both of these values, it is necessary to analyze polledness in Merino sheep separately for the two sexes and not as a combined dataset. A simple combined analysis will dilute the differences observed between sexes. This is reflected in the analyses with the true simulated causative mutation, where the GPI for the combined analyses is the lowest observed (67%).

In previous studies by Dolling [1, 26, 27] in Merino sheep, scurs or knobs are discussed as closely linked alleles to polledness or were considered identical to the allele causing polledness. The penetrance values show scurs and knobs can be the intermediate phenotype between horns and polledness assuming one common genetic mechanism. A similar mechanism has been proposed in cattle where scurs and horned/polledness have been described as two separate traits [28], but a recent study by Wiedemar et al. [29] showed that all scurred animals in their study were heterozygous for one of the polled mutations.

Given the penetrance values per sex for Merino sheep we were able to better understand why prediction of the phenotype is not 100% accurate [9], and we are now better able to understand the underlying mechanism. Further functional studies would need to be conducted to investigate the influence of sex hormones in males and females on the development of horns. In the absence of a good model organism, this will be challenging given the relatively small investments in the sheep industry for fundamental questions like the development of horns.

## Conclusions

We have clearly shown different penetrance values for males and females for horn status. Genotype probabilities from a segregation model, which assumed a monogenic inheritance with incomplete penetrance for horn status, were more significantly associated with the most significant SNP from the Ovine Infinium® HD chip compared to using real phenotypes. GWAS fitting the expected allele count gave a much better fit than GWAS using real phenotypes. Functional studies are needed to investigate whether differences in levels of sex hormones cause differences in horn status and whether more mutations in the *RXFP2* region are present which alter the transcription with a possible influence on horn development. In addition, we have demonstrated that segregation analysis of a trait with mutually exclusive phenotypes, non-additive inheritance, and/or incomplete penetrance can lead to considerably more power in a GWAS. This is because the linearized genotype probabilities analyzed are additive by definition, and they accommodate any incomplete penetrance involved.

## Supplementary Information

Additional file 1: Figure S1. Genome-wide association study for horned/non-horned in Merino sheep, adjusted from [9]. (a) Males, horned/non-horned, (b) Females horned/non-horned, (c) Males and females horned/non-horned. The x-axis indicates the genomic location of the SNPs and each chromosome is color-coded. The y-axis shows the –log10(p-value) of the association statistics for each SNP.

Additional file 2: Figure S2. Genome-wide association study for polled/non-polled in Merino sheep, adjusted from [9]. (a) Males, polled/non-polled, (b) Females polled/non-polled, (c) Males and females polled/non-polled. The x-axis indicates the genomic location of the SNPs and each chromosome is color-coded. The y-axis shows the –log10(p-value) of the association statistics for each SNP.

## Data Availability

Access to phenotypic and pedigree data and simulation code can be arranged upon request to the corresponding author.
