# Supplementary figures and images for: Segregation GWAS to linearize a non-additive locus with incomplete penetrance: an example of horn status in sheep
Source: Genet Sel Evol. 2024 Sep 3;56:61. doi: 10.1186/s12711-024-00928-0 (PMC11373182; doi:10.1186/s12711-024-00928-0)

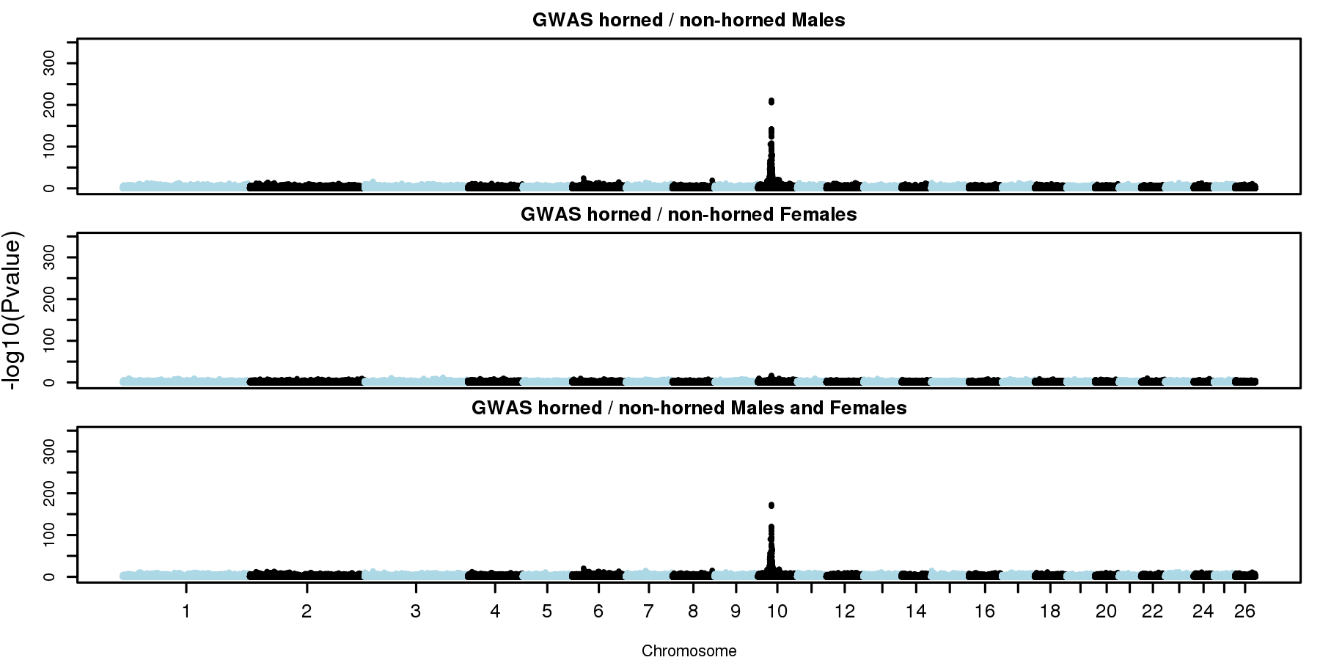

Supplement: Supplementary file 1 — Additional file 1: Figure S1. Genome-wide association study for horned/non-horned in Merino sheep, adjusted from [9]. (a) Males, horned/non-horned, (b) Females horned/non-horned, (c) Males and females horned/non-horned. The x-axis indicates the genomic location of the SNPs and each chromosome is color-coded. The y-axis shows the –log10(p-value) of the association statistics for each SNP. [file 12711_2024_928_MOESM1_ESM.docx]

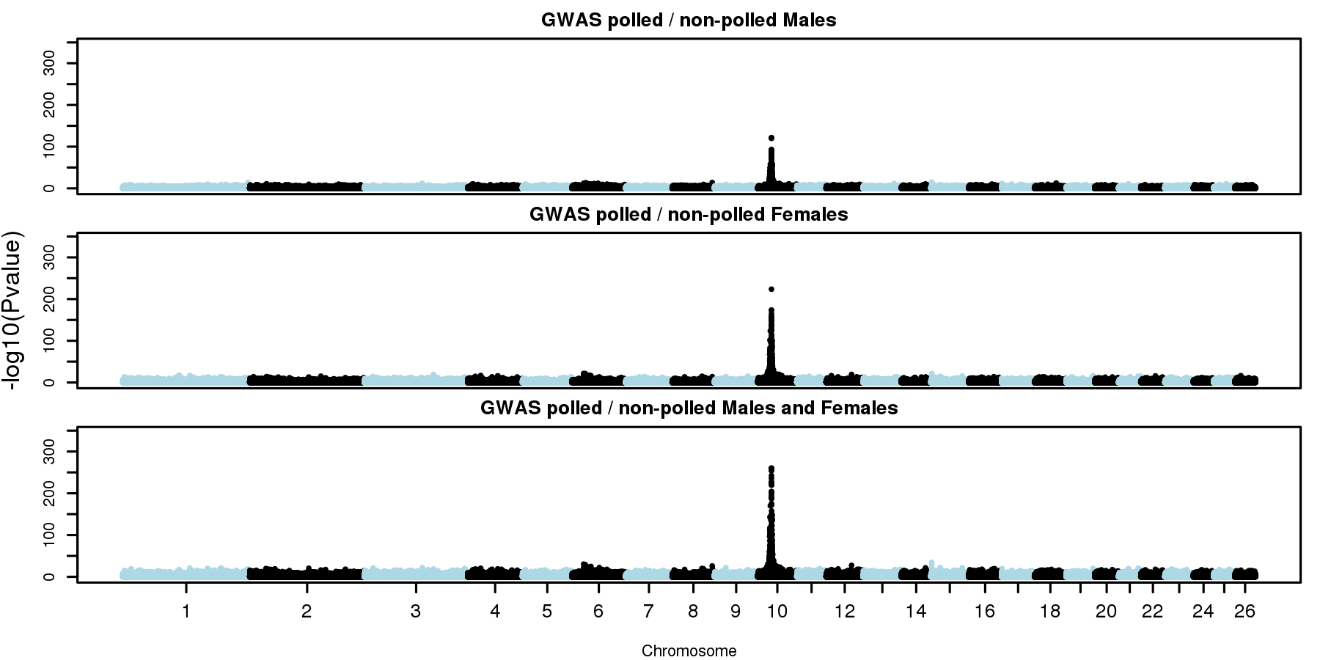

Supplement: Supplementary file 2 — Additional file 2: Figure S2. Genome-wide association study for polled/non-polled in Merino sheep, adjusted from [9]. (a) Males, polled/non-polled, (b) Females polled/non-polled, (c) Males and females polled/non-polled. The x-axis indicates the genomic location of the SNPs and each chromosome is color-coded. The y-axis shows the –log10(p-value) of the association statistics for each SNP. [file 12711_2024_928_MOESM2_ESM.docx]
